# Diclofenac-Derived Hybrids for Treatment of Actinic Keratosis and Squamous Cell Carcinoma

**DOI:** 10.3390/molecules24091793

**Published:** 2019-05-09

**Authors:** Silvia Tampucci, Sara Carpi, Maria Digiacomo, Beatrice Polini, Stefano Fogli, Susi Burgalassi, Marco Macchia, Paola Nieri, Clementina Manera, Daniela Monti

**Affiliations:** 1 Department of Pharmacy, University of Pisa, Via Bonanno 6, 56126 Pisa, Italy; silvia.tampucci@unipi.it (S.T.); sara.carpi@unipi.it (S.C.); b.polini@studenti.unipi.it (B.P.); susi.burgalassi@unipi.it (S.B.); marco.macchia@unipi.it (M.M.); Paola.nieri@unipi.it (P.N.); clementina.manera@unipi.it (C.M.); daniela.monti@unipi.it (D.M.); 2 Interdepartmental Research Center “Nutraceuticals and Food for Health” (NutraFood), University of Pisa, 56126 Pisa, Italy; 3 Department of Clinical and Experimental Medicine, University of Pisa, Via Savi 10, 56126 Pisa, Italy; stefano.fogli@unipi.it

**Keywords:** diclofenac, hybrid, squamous cell carcinoma, actinic keratosis, antiproliferative activity, nanomicelles, in vitro skin permeation/penetration

## Abstract

In this work, hybrid compounds **1**–**4** obtained by conjugation of the non-steroidal anti-inflammatory drug diclofenac, with natural molecules endowed with antioxidant and antiproliferative activity were prepared. The antiproliferative activity of these hybrids was evaluated on immortalized human keratinocyte (HaCaT) cells stimulated with epidermal growth factor (EGF), an actinic keratosis (AK) model, and on human squamous cell carcinoma (SCC) cells (A431). Hybrid **1** presented the best activity in both cell models. Self-assembling surfactant nanomicelles have been chosen as the carrier to drive the hybrid **1** into the skin; the in vitro permeation through and penetration into pig ear skin have been evaluated. Among the nanostructured formulations tested, Nano3Hybrid20 showed a higher tendency of the hybrid **1** to be retained in the skin rather than permeating it, with a desirable topical and non-systemic action. On these bases, hybrid **1** may represent an attractive lead scaffold for the development of new treatments for AK and SCC.

## 1. Introduction

Non-melanoma skin cancer (NMSC) represents the most common cancer among white-skinned people. The term NMSC refers to keratinocyte cancer and essentially to two types of cancer: basal cell carcinoma (BCC) and squamous cell carcinoma (SCC). BCC is characterized by local tissue damage and seldom leads to metastatization while SCC can recur and metastasize. The step that often precedes the onset of this latter tumoral form is called actinic keratosis (AK), that occurs on sun-exposed skin and may progress to invasive SCC in up to 10% of cases [1]. The treatment of AK and SCC includes invasive approaches (excisional surgery, laser ablation, cryosurgery, curettage and electrodessication), and non-invasive approaches (medical and photodynamic therapy). Specifically, when surgical treatment is not feasible, topical pharmacotherapy can be a viable alternative. Diclofenac, a nonsteroidal anti-inflammatory drug, is the most commonly used topical agent to treat AK, due to its effectiveness, poor side effects, tolerability and low cost [2]. Topical diclofenac, effective use for NMSC, is also reported in recent clinical trials [3,4]. Another therapeutic approach, especially for AK, may derive from the protection of skin keratinocytes from UV-induced damages. In fact, the link between sun exposure and AK skin lesions is well-recognized. Excessive exposure to UV radiations may cause mutations in keratinocytes, in particular, UV-A and UV-B radiations induce production of reactive oxygen species (ROS), provoking DNA damage and abnormal cell proliferation. It has been reported that the topical use of antioxidants contributes to decreasing the oxidative damage mediated by UV radiation [5]. Furthermore, natural molecules, known as antioxidants, have recently showed antiproliferative activity in different types of cancer [6,7,8,9]. In particular, hydroxytyrosol (HT) is one of the most powerful antioxidant compounds [10]. Moreover, HT induces apoptosis and inhibits tumor growth in different in vitro and in vivo tumor models [11,12,13]. The structural analogue tyrosol (T), despite a lower antioxidant action than HT, possesses a protective effect on oxidative lesion in cellular models, boosting intracellular antioxidant defense systems. Particularly interesting is its action against H_2_O_2_ induced damage on DNA [14].

In recent years the increasing incidence and morbidity of SCC patients has directed the research towards design of new drug therapies. The multi target approach is considered innovative in drug discovery with encouraging results in various therapeutic fields, especially in the treatment of cancer. The approach of “dual action drugs” in which two or more pharmacophoric portions are incorporated in a single molecule, may allow for obtaining a hybrid with improved efficacy and/or minor side effects than the native drugs [15].

**Figure 1:**
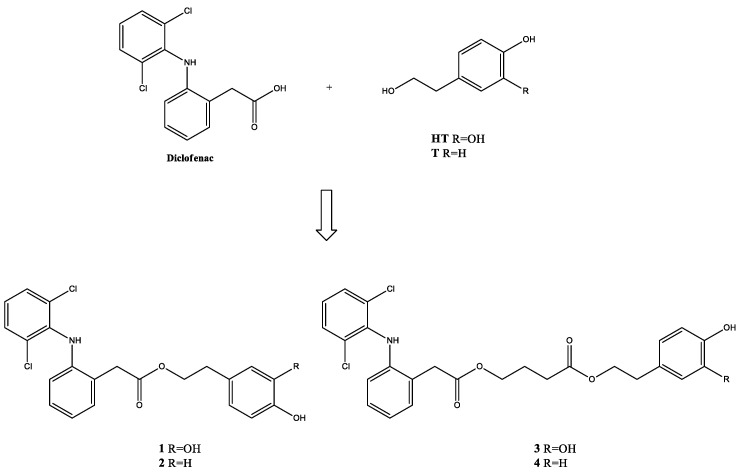
Chemical structure of synthesized hybrids (**1**–**4**).

Based on these findings, in the current study we conjugated the molecule of diclofenac (Figure 1) with the natural antioxidant and antiproliferative compounds, HT and T, (Figure 1) obtaining the hybrids **1** and **2**. Furthermore, we synthesized compounds **3** and **4** characterized by to a linker between the two native compounds.

Hybrids **1**–**4** were tested in AK and in SCC models in order to evaluate their chemopreventive and antitumor activity, respectively. Since the main target of AK treatment is the epidermis, in particular the basal layer and in a major extent the granular and cornified layers, we studied for hybrid 1, that showed the best activity in AK model, a nanostructured drug delivery system as a formulation strategy to selectively direct the drug to a specific site (different skin layers) by enhancing skin penetration while reducing transcutaneous permeation and consequently systemic side effects [16]. Delivery of drugs using nanotechnologies, not only can improve drug stability, but also can reduce skin irritation by avoiding direct contact of the drug with the skin′s surface. The nanomicelles have recently attracted a great deal of attention for drug delivery into the skin for the treatment of dermatological diseases [17,18,19] and in the present research surfactant nanomicelles, able to self-assemble above the critical micelle concentration (CMC), have been chosen as the carrier to drive the hybrid **1** into the skin. The in vitro permeation through and penetration into pig ear skin, a widely accepted model for human skin [20], have been evaluated.

## 2. Results and Discussion

### 2.1. Synthesis

**Scheme 1:**
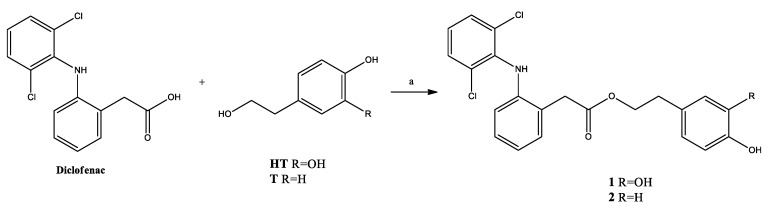
Reagents and conditions: (a) DCC, DMAP, THF, rt, 12 h.

Compounds **1** and **2** were prepared by condensation of diclofenac with HT or T, in the presence of *N*,*N*′-dicyclohexylcarbodiimide (DCC) and a catalytic amount of 4-dimethylaminopyridine (DMAP) in THF at room temperature, as described in Scheme 1.

**Scheme 2:**
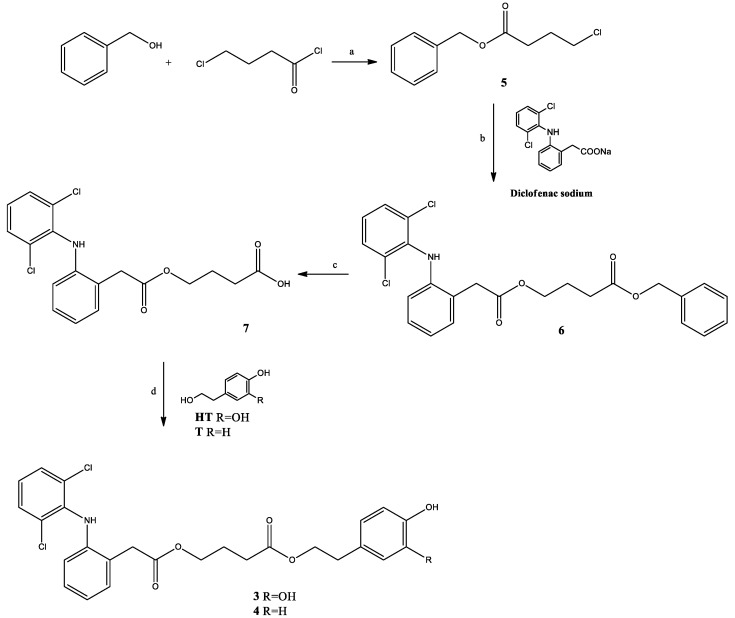
Reagents and conditions: (a) pyridine, toluene, rt, 20 h; (b) DMF, 110 °C, 3 h; (c) H2, Pd/C, EtOH, rt, 3 h; (d) DCC, THF, refluxed, 3 h.

The synthesis of compounds **3** and **4** is shown in Scheme 2. The intermediate **5** prepared by reaction between 4-chlorobutanoyl chloride and 2-phenylmetanol in the presence of pyridine, was condensed with diclofenac sodium obtaining derivative **6**. The subsequent catalytic hydrogenation gave acid **7**, which was submitted to condensation with HT or T using DCC as condensing reagent, afforded compounds **3** and **4**.

### 2.2. Pharmacological Activity

Hybrids **1**–**4** and native compounds (diclofenac, HT, T) were tested to evaluate cytotoxic activity on epidermal growth factor (EGF)-stimulated HaCaT cells as in vitro model of AK.

**Figure 2:**
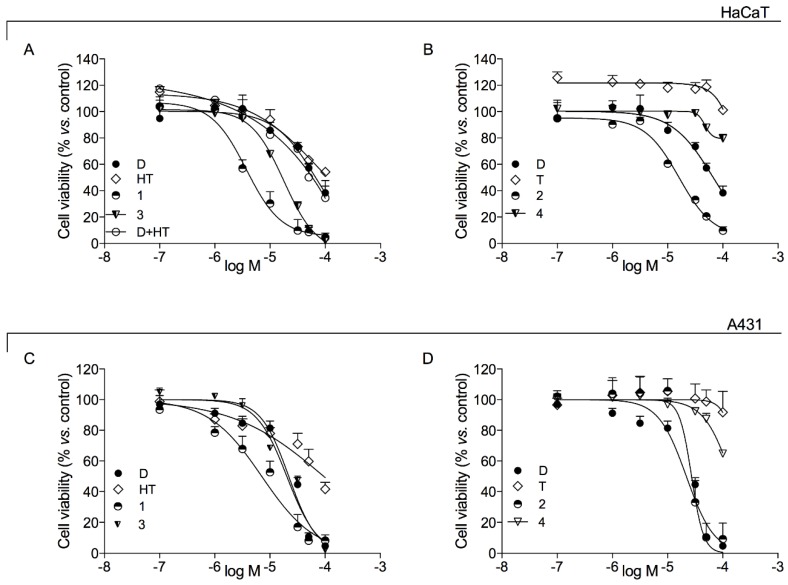
Effects induced by test compounds on cell viability of HaCaT cells after stimulation with 5 ng/mL EGF (**A**, **B**) and A431 cells (**C**, **D**). Cells were exposed to compounds for 72 h and cell viability assessed by neutral red assay. Data are expressed as means ± SEM of three independent experiments, each performed in triplicate. Each hybrid was compared to single agents. D: diclofenac, HT: hydroxytyrosol, T: tyrosol.

The results showed that diclofenac induced a dose-dependent cytotoxic effect with an IC_50_ of 67.07 ± 1.94 µM (Figure 2A,B, Table 1). This effect is in accordance with antiproliferative activity showed by diclofenac itself in other cancer models [21,22,23,24]. A cell viability decrease was also observed with HT (IC_50_ 72.31 ± 3.58 μM, Figure 2A, Table 1). This was not surprising, since this phenolic phytochemical, in addition to the antioxidant property, has already been shown to contrast cell viability in different cancer types [11,13,25,26,27,28,29,30] as well as to down-regulate the EGF receptors on colon cancer cells [31]. The hybrids **1** and **3**, in which diclofenac is conjugated to HT directly (hybrid **1**) or through a 3C linker (hybrid **3**), showed, a dose-dependent cell viability decrease with an IC_50_ value of 3.71 ± 1.08 µM and 17.77 ± 1.09 µM (Figure 2A, Table 1), respectively. In particular, the hybrid **1** induced a cell viability decrease with a significantly higher potency (about 20-fold) than those of native drugs, diclofenac and HT (Figure 2A, Table 1). The association of diclofenac and HT, at the same concentration in the hybrids, induced a not significant decrease on cell viability compared to the individual treatments (Figure 2A). These data suggested the potential advantage of hybrids **1** and **3** compared to the association of single treatments.

As regards T, an IC_50_ value was not reached in the concentration range evaluated (Figure 2B, Table 1) in our experiments. This data is in agreement with a reduced efficacy of T in comparison to HT on tumor proliferation and EGF receptors down-regulation, as reported previously by Terzuoli et al. on colon cancer [31]. Diclofenac-T hybrid **2** was 4-fold more potent than diclofenac, with an IC_50_ of 16.06 ± 1.15 µM (Figure 2B, Table 1). On the contrary, the analogue hybrid **4**, which differs from **2** for the presence of the 3C linker, showed an antiproliferative effect lower than that of diclofenac (Figure 2B, Table 1).

Comparing the IC_50_ values of the hybrids **1** and **2** (in which diclofenac is directly linked to HT or T, respectively) with those of the hybrids **3** and **4,** characterized by a linker between the two native molecules, it is possible to show that the presence of linker has a negative effect for the cytotoxic activity of hybrids on the AK cell model.

Hybrids **1**–**4** and native compounds (diclofenac, HT, T) were tested, also, to evaluate their cytotoxic activity on squamous carcinoma A431 cells line (Figure 2B,C, Table 1).

In these cells, diclofenac induced a dose-dependent cytotoxic effect with an IC_50_ of 31.96 ± 1.05 µM (Table 1). This result is in line with diclofenac activity previously reported [21,22,23,24,32,33,34]. The activity of HT in A431 cells was reduced compared to EGF-stimulated HaCaT cells (Figure 2C). This behavior could be explained considering that EGF inhibits cell growth in A431 cells, while stimulate growth in HaCaT cells [31,35] and that HT is an EGF receptor deregulator. In A431 cells, hybrid **1** (diclofenac-HT) was the most powerful cytotoxic agent (IC_50_ 13.51 ± 2.49 µM) compared to other hybrids and the native molecules diclofenac (31.96 ± 1.05 µM) and HT, although its IC_50_ value was about 4 fold greater than that obtained in EGF-stimulated HaCaT cells (Figure 2C, Table 1). The analogue hybrid **3** showed minor activity compared to hybrid **1** also in this SCC model.

T showed a reduced anticancer activity in comparison to HT as above reported for EGF-stimulated HaCaT cells (Figure 2D). This result is in line with evidences obtained in colon cancer [31]. As regards compound **2**, where HT was substituted by T, its antitumor effect is reduced compared to compound **1**. Hybrid **2** showed an IC_50_ value (27.51 ± 1.24 µM) without a significant difference with respect to diclofenac (31.96 ± 1.05 µM) (Figure 2D, Table 1). The hybrid **4** was not able to induce modification in cell viability in SCC as well as in AK model (Figure 2D, Table 1). Additionally, in A431 cell line, as in EGF-stimulated HaCaT cells, the presence of linker as in compounds **3** and **4**, is deleterious for antiproliferative activity.

### 2.3. Nanomicellar Formulation Containing Hybrid ***1***

Hybrid **1**, selected for the best activity in both cell models, was formulated in a nanostructured drug delivery system. The prepared self-assembling nanomicelles consisted of a mixture of two non-ionic surfactants with the same HLB value (13 and 13.2 for Vit-E TPGS and OPEE, respectively). In particular, Vit-E TPGS was chosen in virtue of its multivalued features as excellent solubilizer and bioavailability enhancer of hybrophobic molecules, as well as to act as selective antitumor agent inducing apoptosis in cancer cells and not healthy ones. Moreover its ability to inhibit the activity of P glycoprotein makes it an important excipient to overcome multidrug resistance that represents an impediment to the success of anticancer therapy [36,37,38]. Nanomicellar formulations containing hybrid **1** were characterized from the physico-chemical point of view. The results in term of size and size distribution, entrapment efficiency and drug loading related to the formulations under study, are summarized in Table 2. The nanomicelles size ranged between 14 and 70 nm with a narrow distribution (0.2 < polidispersivity index < 0.36). The dimension of nanomicelles is a crucial aspect that affects drug permeation and distribution into the skin [16]. It is noteworthy that the size of micelles can range from 10 to 200 nm and can be increased by drug incorporation. Their mechanism of action is not clearly demonstrated but it is known that they can penetrate through stratum corneum and accumulate in hair follicles. Moreover it has been observed skin penetration into deeper skin layers followed by drug delivery in the intercluster region, one of the penetration pathways through the skin [19]. All of the prepared nanomicellar formulations had a good drug efficiency in the range between 79% and 96%. It can be noted that for the same total amount of surfactants (3%), the size appeared to increase with increasing the drug loading.

Hybrid loaded nanomicellar formulations were subjected to in vitro studies of permeation through and penetration into pig ear skin.

The results of the in vitro experiments are summarized in Table 3, where the relevant permeation parameters (flux, lag time, amount of drug permeated after 24 h) are reported for each formulation. Data are shown as diclofenac in the case of Solaraze or as both hybrid **1** and diclofenac (derived from hydrolysis of hybrid **1**) for the nanomicellar formulations. In all cases, nanomicellar formulations consisting of 3% of total amount of surfactants did not produce any permeation of the hybrid **1** while, after a lag time, it was possible to detect in the receiving phase the presence of diclofenac as hydrolysis product of hybrid **1**.

Hybrid **1** did not permeate across the skin, maybe because of its high molecular weight (432.30 g/mol), while the native drug diclofenac permeated the skin with a transdermal flux ranging from 0.04 to 0.58 nmol/cm^2^·h. Nonetheless, the amount of diclofenac permeated was definitely lower than that obtained by the reference, considering also the differences in the drug content of the formulations. The lipophilic nature of diclofenac sodium (partition coefficient *n*-octanol/water, log POW = 4.98) appeared to promote a better interaction with the different skin layers, in particular with the stratum corneum, known as the main barrier to permeation.

Nanomicelles containing 5% of total amount of surfactant (Nano5Hybrid30) produced a permeation of both hybrid **1** and diclofenac derived from its hydrolysis. The higher amount of surfactant appeared to increase the skin permeability, promoting the transcutaneous permeation, as well known [39,40].

**Figure 3:**
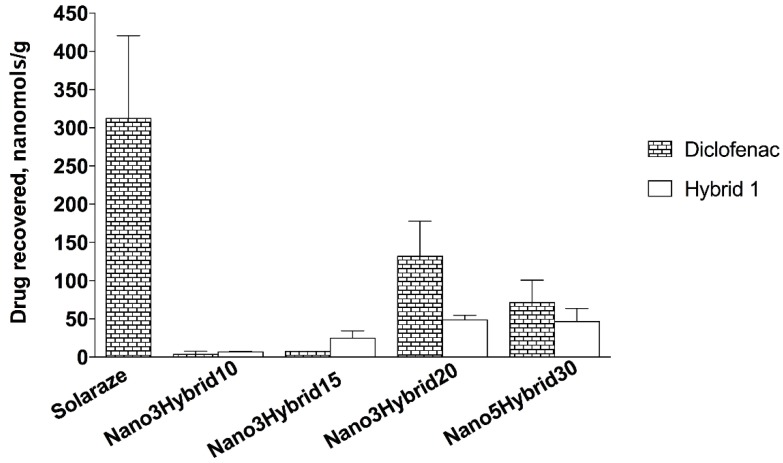
Drug recovered in the pig ear skin after application of the formulation under study (nanomols/g of skin, mean ± SEM, *n* = 6).

The skin recovery results after application of the different formulation under study are shown in the histogram reported in Figure 3. Drug recovered in the pig ear skin as diclofenac or hybrid **1** compound is reported in nanomols/g of skin. When the different dispersions of hybrid **1** were used as donor phase, both hybrid **1** and diclofenac derived from the hydrolysis of hybrid were found. The reference Solaraze gel produced a high recovery of drug in the skin. In the case of Nano3Hybrid formulations, the amount of drug in form of hybrid and diclofenac increased proportionally to the hybrid **1** content in the formulation. Nano3Hybrid20 appeared the best nanomicellar formulation, even compared to Nano5Hybrid30 formulation, which, in term of recovery, did not give any advantage (117.7 ± 15.21 vs. 180.4 ± 36.68 nmols/g, for Nano5Hybrid30 and Nano3Hybrid20, respectively).

The results of the comparison of the different formulations under study in terms of permeation and recovery of drug (as diclofenac and/or hybrid **1**) are summarized in Table 4. Solaraze produced high drug accumulation with a relevant permeation; Nano3Hybrid formulations appeared to promote the recovery of both hybrid **1** and diclofenac as hydrolysis product of Hybrid (both therapeutically active) and the recovery increased with increasing the Hybrid amount encapsulated in the nanomicelles. Moreover, they did not facilitate the transcutaneous permeation. Nano3Hybrid20 appeared the best formulation, to be preferred also to Nano5Hybrid30, which, next to a non-high recovery, allowed a cutaneous permeation of both drugs involved.

**Figure 4:**
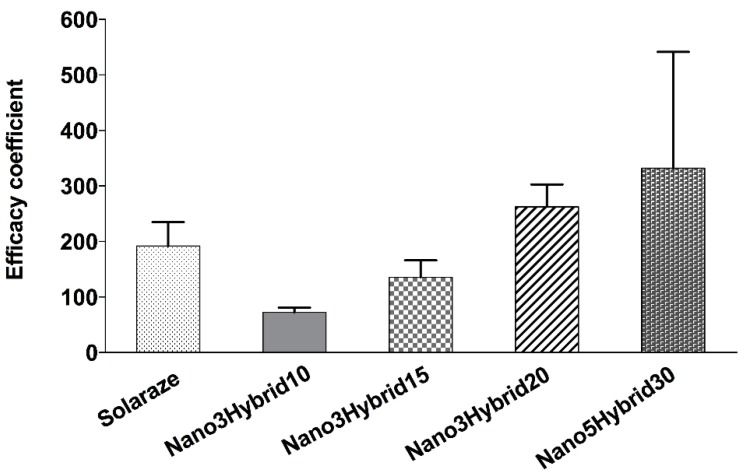
Efficacy of the drug retained in the skin related to its capacity to hinder the growth of EGF-stimulated HaCaT cells.

Finally, in the Figure 4, the relationship between drug recovered in the skin and the relative activity on EGF-stimulated HaCaT cells is illustrated. The histogram highlights the efficacy of the drug (as diclofenac for Solaraze and as diclofenac plus hybrid **1** for nanomicellar formulations) retained in the skin related to the capacity of these drugs to hinder the growth of EGF-stimulated HaCaT cells, as AK model. It is evident the greater efficacy of hybrid **1** encapsulated in Nano3 and Nano5 formulations with respect to the reference, even if with no-statistically significant differences. It is noteworthy that in the case of nanomicellar formulations the amount of drug applied on the skin (19.3 and 27.01 μmols/mL for Nano3Hybrid20 and Nano5Hybrid30, respectively) was 3 to 5-fold lower than that of Solaraze (94.2 μmols/mL). Since hybrid **1** had much greater activity than diclofenac (about 20-fold) demonstrated by the IC_50_ value (67.07 ± 1.94 vs. 3.71 ± 1.08 μM, for diclofenac and hybrid **1**, respectively), it appears evident that hybrid **1** recovered on the skin was enough to have the same or more activity than the commercial product.

## 3. Materials and Methods

### 3.1. Chemicals and Instrumentation

Commercially available reagents were purchased from Sigma Aldrich or TCI, and used without purification.

To prepare nanomicelles the following materials were used: octylphenoxy poly(ethyleneoxy)ethanol (OPEE, IGEPAL^®^ CA-630, Sigma-Aldrich, Milan, Italy); d-α-Tocopherol polyethylene glycol succinate (Vit E-TPGS, Kolliphor^®^TPGS, BASF, Ludwigshafen, Germany).

^1^H-NMR and ^13^C-NMR spectra were recorded on a Bruker AVANCE III™ 400 spectrometer (Bruker Corporation, Billerica, MA, USA, operating at 400 MHz). Chemical shift (δ) were reported in parts per million (ppm) related to the residual solvent signal, coupling constants (*J*) are expressed in Hertz (Hz). Evaporation was carried out in vacuo using a rotating evaporator. Silica gel flash chromatography was performed using silica gel 60 Å (0.040–0.063 mm; Merck, Milan, Italy). Reactions were monitored on TLC on Merck aluminium silica gel (60 F254) plates that were visualized under a UV lamp (λ = 254 nm). Melting points were determined on a Kofler hot-stage apparatus and are uncorrected.

All synthesized compounds were analyzed by HPLC, showing a purity ≥95%. A Bechman HPLC instrument equipped with a System Gold Solvent Delivery module (Pumps) 125, System Gold UV/VIS Detector 166, Detector set to 278 nm was employed. Analyses were performed on a reverse phase C18 column (Phenomenex 250 × 4.6 mm, 5 mm particle size, Gemini, Phenomenex, Torrance, CA, USA). The mobile phase was constituted by a mixture of H_2_O/AcOH (0.1% *v*/*v*) (eluent A) and ACN (eluent B) on isocratic elution with 70% of B. The flow rate was 0.8 mL/min.

### 3.2. Synthesis Method

#### 3.2.1. General Procedure for the Synthesis of Compounds **1**, **2**

To a solution of HT or T (0.84 mmol) in THF (2 mL), diclofenac (250 mg, 0.84 mmol), DCC (209 mg, 1.012 mmol) and DMAP (8 mg, 0.06 mmol) were added. The resulting mixture was stirred at room temperature for 24 h, then the suspension was filtered and the solution was evaporated. The crude product obtained was purified as described below.

*3,4-Dihydroxyphenethyl 2-(2-((2,6-dichlorophenyl)amino)phenyl)acetate* (**1**). Purified by flash chromatography on a silica gel column, eluting with ethyl acetate/petroleum ether (3:7). Yield 52%; white solid mp 123–125 °C. ^1^H-NMR (CDCl_3_): δ (ppm) 7.34 (d, 2H, *J* = 8.0 Hz, Ar), 7.22 (dd, 1H, *J* = 1.2, 7.6 Hz, Ar), 7.15 (dt, 1H, *J* = 1.2, 7.6 Hz, Ar), 6.95–7.01 (m, 2H, Ar), 6.80 (br s, 1H, NH), 6.73 (d, 1H, *J* = 8.0 Hz, Ar), 6.53–6.58 (m, 3H, Ar), 5.01 (br s, 1H, OH), 4.91 (br s, 1H, OH), 4.31 (t, 2H, *J* = 6.8 Hz, CH_2_), 3.80 (s, 2H, CH_2_Ph), 2.82 (t, 2H, *J* = 6.8 Hz, CH_2_). ^13^C-NMR (CDCl_3_): δ (ppm) 172.45, 143.45, 142.95, 142.48, 137.78, 131.21, 130.48, 129.81, 129.01, 128.09, 124.35, 122.04, 121.56, 118.25, 116.07, 115.50, 66.15, 38.86, 34.50. HPLC analysis: retention time = 7.50 min; peak area, 96% (280 nm).

*3-Hydroxyphenethyl 2-(2-((2,6-dichlorophenyl)amino)phenyl)acetate* (**2**). Purified by chromatography on a silica gel column, eluting with ethyl acetate/petroleum ether (1:9). Yield 30%; yellow solid mp 120–122 °C; ^1^H-NMR (CDCl_3_): δ (ppm) 7.34 (d, 2H, *J* = 8.0 Hz, Ar), 7.21 (dd, 1H, *J* = 1.2, 7.6 Hz, Ar), 7.13 (dt, 1H, *J* = 1.6, 7.6 Hz, Ar), 7.00 (d, 2H, *J* = 8.4 Hz, Ar), 6.94–7.00 (m, 2H, Ar), 6.84 (br s, 1H, NH), 6.71 (d, 2H, *J* = 8.4 Hz, Ar), 6.55 (d, 1H, *J* = 7.6 Hz, Ar), 4.60 (br s, 2H, OH), 4.31 (t, 2H, *J* = 7.1 Hz, CH_2_), 3.79 (s, 2H, CH_2_Ph), 2.88 (t, 2H, *J* = 7.1 Hz, CH_2_). ^13^C-NMR (CDCl_3_): δ 172.32, 154.37, 142.89, 137.99, 131.06, 130.50, 130.36, 130.21, 129.85, 129.75, 129.01, 128.11, 124.47, 124.18, 122.15, 118.40, 115.51, 66.08, 38.83, 34.37. HPLC analysis: retention time = 10.43 min; peak area, 99% (280 nm).

#### 3.2.2. General Procedure for the Synthesis of Compounds **3**, **4**

To a solution of compound **7** (200 mg, 0.52 mmol) in THF (2 mL) was added DCC (129 mg, 0.628 mmol) and HT or T (0.52 mmol). The reaction mixture was stirred at reflux for 3 h, then the solid were filtered out and the solvent was evaporated. The crude product obtained was purified as described below.

*3,4-Dihydroxyphenethyl 4-(2-(2-((2,6-dichlorophenyl)amino)phenyl) acetoxy)butanoate* (**3**). The residue was purified by chromatography on a silica gel column, eluting with CHCl_3_/AcOEt 7:3, and subsequently triturated with Et_2_O. Yield 20%; oil. ^1^H-NMR (CDCl_3_): δ (ppm) 7.35 (d, 2H, *J* = 8.0 Hz, Ar), 7.22 (dd, 1H, *J* = 1.2, 7.2 Hz, Ar), 7.11–7.16 (m, 1H, Ar); 6.99 (t, 1H, *J* = 8.0 Hz, Ar), 6.97 (t, 1H, *J* = 7.4 Hz, Ar), 6.82 (br s, 1H, NH), 6.78 (d, 1H, *J* = 8.0 Hz, Ar), 6.72 (d, 1H, *J* = 2.0 Hz, Ar) , 6.62 (dd, 1H, *J* = 2.0, 8.0 Hz, Ar), 6.56 (d, 1H, *J* = 8.0 Hz, Ar), 5.26 (br s, 1H, OH), 5.75 (br s, 1H, OH), 4.25 (t, 2H, *J* = 6.6 Hz, CH_2_), 4.15 (t, 2H, *J* = 6.6 Hz, CH_2_), 3.82 (s, 2H, CH_2_Ph), 2.81 (t, 2H, *J* = 6.6 Hz, CH_2_), 2.36 (t, 2H, *J* = 7.0 Hz, CH_2_), 1.92–2.01 (m, 2H, CH_2_). ^13^C-NMR (CDCl_3_): δ 172.98, 172.89, 143.86, 142.79, 142.70, 137.88, 131.00, 130.55, 129.63, 129.01, 128.21, 124.28, 124.23, 122.24, 121.23, 118.47, 116.09, 115.51, 65.49, 64.59, 38.66, 34.49, 30.75, 24.04. HPLC analysis: retention time = 11.47 min; peak area, 95% (280 nm).

*4-Hydroxyphenethyl 4-(2-(2-((2,6-dichlorophenyl)amino)phenyl)acetoxy)butanoate* (**4**). Purified by chromatography on a silica gel column, eluting with CHCl_3_/AcOEt 9:1, and subsequently triturated with Et_2_O. Yield 23%; oil. ^1^H-NMR (CDCl_3_): δ (ppm) 7.34 (d, 2H, *J* = 8.0 Hz, Ar), 7.21–7.24 (m, 1H, Ar), 7.23 (d, 2H, *J* = 8.4 Hz, Ar), 7.13 (dt, 1H, *J* = 1.5, 7.7 Hz, Ar), 7.02 (d, 2H, *J* = 8.4 Hz, Ar), 6.94–7.03 (m, 2H, Ar), 6.91 (br s, 1H, NH), 6.56 (d, 1H, *J* = 7.6 Hz, Ar), 4.27 (t, 2H, *J* = 6.4 Hz, CH_2_), 3.85 (t, 2H, *J* = 6.4 Hz, CH_2_), 3.83 (s, 2H, CH_2_Ph), 2.86 (t, 2H, *J* = 6.6 Hz, CH_2_), 2.63 (t, 2H, *J* = 7.4 Hz, CH_2_), 2.08–2.15 (m, 2H, CH_2_). ^13^C-NMR (CDCl_3_): δ (ppm) 172.45, 171.52, 149.27, 142.81, 137.90, 136.31, 130.95, 130.10, 129.61, 128.98, 128.15, 124.34, 124.15, 122.17, 121.64, 118.43, 64.22, 63.65, 38.66, 30.87, 24.08. HPLC analysis: retention time = 11.35 min; peak area, 95% (280 nm).

#### 3.2.3. Synthesis of Benzyl 4-Chlorobutanoate (**5**)

To a solution of phenylmethanol (0.77 g, 7.09 mmol) in toluene anhydrous (1 mL), pyridine (0.56 g, 7.09 mmol) and 4-chlorobutanoyl chloride (1.00 g, 7.09 mmol) were added. The reaction mixture was stirred at room temperature overnight, then H_2_O was added and the toluene was evaporated. The resulting aqueous phase was extracted with Et_2_O, the organic phase was washed with 0.5 M HCl and a saturated NaHCO_3_ solution. The organic phase was then dried, filtered and evaporated to give compound **5** (1.28 g, 6.02 mmol, 85% yield), which was used for the subsequent reaction without further purification. ^1^H-NMR (CDCl_3_): δ 7.31–7.39 (m, 5H, Ar), 5.13 (s, 2H, CH_2_), 3.60 (t, 2H, *J* = 6.4 Hz, CH_2_), 2.56 (t, 2H, *J* = 7.2 Hz, CH_2_), 2.08–2.15 (m, 2H, CH_2_).

#### 3.2.4. Synthesis of Benzyl 4-(2-(2-((2,6-Dichlorophenyl)amino)phenyl)acetoxy)butanoate (**6**)

Compound **5** (1.09 g, 5.13 mmol) was added to a solution of sodium diclofenac (1.63 g, 5.13 mmol) in DMF (4.0 mL) heated to 110 °C. Stirring was continued at the same temperature for about 3.5 h. After this period, the reaction mixture was cooled at room temperature and H_2_O was added. The aqueous phase was extracted with Et_2_O, and then the organic phase was dried, filtered and evaporated. The residue which was purified by flash chromatography eluting with hexane/AcOEt 9:1, providing compound **6** (848 mg, 1.79 mmol, 35% yield). ^1^H-NMR (CDCl_3_): δ (ppm) 7.32–7.36 (m, 7H, Ar), 7.21 (dd, 1H, *J* = 1.2, 7.6 Hz, Ar), 7.12 (dt, 1H, *J* = 1.5, 7.8 Hz, Ar), 6.92–7.00 (m, 2H, Ar), 6.91 (br s, 1H, NH), 6.54 (d, 1H, *J* = 8.0 Hz, Ar), 5.12 (s, 2H, CH_2_Ph), 4.19 (t, 2H, *J* = 6.4 Hz, CH_2_), 3.79 (s, 2H, CH_2_Ph).

#### 3.2.5. Synthesis of 4-(2-(2-((2,6-Dichlorophenyl)amino)phenyl)acetoxy)butanoic acid (**7**)

Pd/C (29 mg) was added to a solution of compound **6** (250 mg, 0.59 mmol) in absolute EtOH (2 mL). This mixture was subjected to catalytic hydrogenation at room temperature for 3 h, then the suspension was filtered on a celite, and the solvent evaporated to obtain compound **7** (195 mg, 0.51 mmol, 86% yield). ^1^H-NMR (CDCl_3_): δ (ppm) 7.34 (d, 2H, *J* = 8.0 Hz, Ar), 7.22 (dd, 1H, *J* = 1.4, 7.4 Hz, Ar), 7.10–7.14 (m, 1H, Ar), 6.98 (t, 1H, *J* = 8.0 Hz, Ar), 6.95 (t, 1H, *J* = 7.4 Hz, Ar), 6.55 (d, 1H, *J* = 8.0 Hz, Ar), 4.17–4.22 (m, 2H, CH_2_), 3.81 (s, 2H, CH_2_Ph), 2.43 (d, 2H, *J* = 7.4 Hz, CH_2_), 1.96–2.04 (m, 2H, CH_2_).

The ^1^H-NMR and ^13^C-NMR spectra of the compounds **1**–**4** are available in supplementary materials.

### 3.3. Biological Assay Procedures

#### 3.3.1. Cell Cultures and Experimental Models

The immortalized human keratinocyte (HaCaT) cells and human squamous carcinoma (A431) cells (Sigma-Aldrich, Milan, Italy) were cultured in RPMI 1640 (Euroclone, Milan, Italy) supplemented with 10% fetal bovine serum (FBS), 100 U/mL penicillin, and 100 μg/mL streptomycin (Euroclone) at 37 °C in a humidified atmosphere containing 5% CO_2_.

HaCaT cells under the proliferative stimulus of EGF were used as model of actinic keratosis (AK), a precancerous disease that can progress in cutaneous squamous cell carcinoma (cSCC), according to the critical role played by the EGFR/Fyn/Src/Erk pathway for promoting cSCC formation [41]. Cells were stimulated with EGF at the concentration 5 ng/mL for whole time of treatment with test compounds (see below).

Both HaCaT (EGF-stimulated) and A431 cells were incubated for 72 h with the test compounds at concentrations in the range 0.1–100 μM.

#### 3.3.2. Cell Viability Assay

Cell viability was measured using the Neutral Red (NR) Assay (N2889, Sigma-Aldrich, Darmstadt, Germany) as previously reported [42]. HaCaT and A431 cell lines were seeded at a density of 5 × 10^4^ cells/well in 10% FBS medium in a 96-well plate. After 24 h, test compounds, dissolved in DMSO, were diluted in sterile culture medium supplemented with EGF (HaCaT cells) or 1% FBS (A431 cells) and added into the well plate. DMSO did not exceed 0.2% *v*/*v* in the culture medium. Diclofenac, HT, T and hybrids **1**–**4** were tested. After 72 h from treatment, NR was added and cells incubated for 2 h. At the end of the incubation period, cells were washed with PBS and, then, incubated for 10 min with a NR solubilisation solution (1% acetic acid in 50% ethanol). The absorbance was measured at 540 nm using the Infinite M200 NanoQuant instrument (Tecan, Salzburg, Austria). Optical density values from vehicle-treated cells were considered as 100% cell viability.

### 3.4. Nanomicellar Formulation Containing Hybrid ***1***

#### 3.4.1. Preparation of self-assembling Surfactant Nanomicelles

Hybrid **1**-loaded self-assembling surfactant nanomicelles (NanoHybrid) were prepared using two non-ionic surfactants, Vit-E TPGS and OPEE (molar ratio 1:1). The two surfactants were heated at 50 °C; then drug was mixed together the other components to obtain a homogeneous blend, finally the water was added and the final mixture was stirred overnight and filtered through cellulose acetate filters (0.22 μm pore size, Minisart^®^ NML Syringe filters, Sartorius, Florence, Italy) to remove unloaded drug, aggregates and other foreign particulates.

Four nanomicellar formulation were prepared, three containing 3.0% of total surfactant amount and one with 5.0% of total surfactants with an increasing amount of drug added from 10 to 30 mM. The composition of the mixtures used for the preparation of hybrid-loaded nanomicelles is reported in Table 5.

#### 3.4.2. Physico-Chemical Characterization

Hybrid **1**- loaded nanomicelles were characterized with respect to size, size distribution and encapsulation efficiency in term of entrapment and drug loading.

Size and size distribution of the nanomicelles were determined by measuring the rate of fluctuations in laser light intensity scattered by the nanomicelles immediately after their preparations by using a Dynamic Light Scattering (DLS) Beckman Coulter^®^ N4 Plus (Beckman Coulter s.r.l, Milan, Italy). The samples were eventually diluted with ultrapure water (MilliQ, Millipore, Merck) previously filtered through 0.45 μm Millipore Polyethersulfone membrane, Millipore Express PLUS, Merck) to an appropriate concentration chosen on the basis of the measurement intensity, which was in the range between 5 × 10^4^ and 1 × 10^6^ counts per second (cps). The average size for each nanomicelles formulation was obtained on three different samples of nanomicelles for which 6 runs were carried out, using an angle of 90° and run time of 200 s at 20 °C.

The entrapment efficiency, defined by the percentage of drug loaded in the core of the nanomicelles with respect to that initially added to the formulation, was determined by HPLC. An aliquot of each Hybrid-loaded nanomicellar formulation was diluted with methanol and the amount of drug was detected by HPLC analysis. The percent entrapment and loading efficiency of hybrid **1** were calculated according to Equations (1) and (2), respectively.

Percent of drug entrapped = (mass of drug in nanomicelles) × 100/(mass of drug added in formulation)(1)

Loading efficacy = (mass of drug in nanomicelles × 100)/(mass of drug added + mass of surfactants in the formulation)(2)

#### 3.4.3. In Vitro Cutaneous Permeation and Distribution Studies

Porcine ears skin, obtained from freshly sacrificed animals in a local slaughterhouse, was used as model. After cleaning with depurated water, full-thickness skin was removed with a scalpel from the cartilage of the outer region. The excised pig ear skin was carefully deprived of the adhering fat and subcutaneous tissue. Prior to the experiments, the pig hairs were abscised and the skin was gently washed. The skin with a thickness about 1.46 ± 0.06 mm was placed in the Gummer-type diffusion cells with the stratum corneum facing the donor compartment and an available diffusion area of 1.23 cm [43,44]. The donor phase consisted of five-hundred microliters of nanomicellar formulations under study and Solaraze^®^ commercial product (Almirall S.A, Barcelona, Spain), hyaluronic acid gel containing 3% diclofenac, used as reference. The receiving phase (5 mL) consisted of deionized water maintained at 37 °C and stirred at 600 rpm. At predetermined time intervals, 5 mL samples of receiving phase were withdrawn for HPLC analysis and replaced with the same volume of fresh fluid. All experiments lasted 24 h and were replicated four times; sink conditions were maintained throughout the entire study.

At the end of the permeation experiments the skin was collected, rinsed with distilled water to eliminate excess of vehicle from the skin surface and gently wiped with cotton wool tampons. In order to extract the drug, the skin was weighed, shredded into small pieces and treated with 2.0 mL of 2% sodium lauryl sulphate (SDS, Sigma-Aldrich, Milan Italy) for 24 h at 37 °C. After treatment with a methanol:chloroform (2:1) solution (3 mL) for 1 h at 37 °C under stirring, the mixture was centrifuged at 4000 rpm for 15 min. Two hundred microliters of supernatant were dried in vacuo and subsequently dissolved in methanol for HPLC analysis. The validation of the extraction procedure was performed as previously described in Monti et al. 2015 [45].

The concentration of diclofenac and hybrid **1** in receiving fluids and in the skin samples was selectively determined by HPLC. The apparatus consisted of a Shimadzu LC-10AD VP system with an UV SPD-10AV VP detector and a CBM-20A interface (Shimadzu). The injection valve was a Rheodyne with a capacity of 20 µL and a Luna^®^ C18 (5 µm; 150 × 4.6 mm) column was employed. The mobile phase consisted of a mixture of acetonitrile:water containing 0.5% glacial acetic acid (70:30) (pH 3.7). The flux was 0.8 mL/min and the detection wavelengths were 254 and 280 nm for diclofenac and hybrid **1** with a retention time of 5.0 and 7.0 min, respectively.

The amount of product under study in the samples was determined by comparison with appropriate standard curves. In the case of skin samples, standard curves were obtained by adding increasing amount of the compounds to blank biological matrix.

Linear regression analysis of pseudo steady state diffusion plots allowed calculation of the following parameters: steady-state flux (J, nmols/cm^2^·h), given by Q/At, where Q is the amount of permeant diffusing across area A in time t; lag time (h), indicating the time taken by the drug to saturate the membrane and reach the receiving phase, calculated from the X-axis intercept values of the regression lines and the amount of drug permeated at the end of the experiment (Q24h, nmols).

Moreover, the extraction procedure allowed calculation of the drug content (nanomols/g skin) recovered in the skin at the end of the permeation studies.

An efficacy coefficient (EC) is reported to compare the biological activity of the drugs involved in this work (diclofenac and Hybrid **1**), taking into account the drug recovered in the biological membrane. EC was calculated as a ratio of the amount of drug retained into the porcine ear skin per unit of weight to IC_50_ value of each drug on EGF-stimulated HaCaT cells, considering the area and thickness of the biological membranes used.

## 4. Statistical Analysis

Data related to biological assay procedures are presented as mean ± standard error of the mean (SEM) from at least three independent experiments. Statistical analysis was performed by one-way ANOVA, followed by Tukey’s post-test for multiple comparisons.

Regarding in vitro cutaneous permeation and distribution studies, data are reported as the mean ± SEM (*n* = 6). Statistical differences between permeation parameters were assessed by GraphPad Prism software (GraphPad Software Inc., San Diego, CA, USA). Group comparison was assessed by using the Student’s two-tailed unpaired t-test. In all cases, differences were considered statistically significant at *p* < 0.05.

## 5. Conclusions

In conclusion, in the last few years, the multi-target approach seems advantageous for the treatment of complex diseases. For this reason, many hybrids for the treatment of different types of pathologies are proposed with encouraging results [15]. However, there are few examples of hybrids used for AK and SCC diseases [46].

In this work, we synthetized compounds (**1**–**4**) obtained by combining diclofenac with T and HT, natural molecules for which antioxidant and antiproliferative activity had been previously described and we evaluated their in vitro activity against AK and SCC models. The hybrid compound **1** has shown a higher antiproliferating activity with respect to native compounds in both AK and SCC models.

Self-assembling nanomicelles have been used as carriers for the delivery of the drug to the site of action and in particular the Nano3Hybrid20 resulted the most effective since it produced a lower amount of drug permeated through the skin with a consequent reduction of systemic side effects. Besides, Nano3Hybrid20 formulation improved an adequate drug accumulation into the cutaneous layers to provide the same therapeutic activity as the commercial reference formulation loaded with an amount of drug about 5-fold higher. The lower amount of drug coming into contact with the skin could contribute to reduce irritative local phenomena, more common for long-term therapy in the precancerous skin lesions.

## Figures and Tables

**Table 1:** IC_50_ mean values ± Standard Error of the Mean (SEM) for test compounds on EGF-stimulated HaCaT and A431 cell lines ^1^.

Treatment	IC_50_ ± SEM (μM)	
	EGF-stimulated HaCaT	A431
D	67.07 ± 1.94	31.96 ± 1.05
HT	72.31 ± 3.58	NR
1	3.71 ± 1.08	13.51 ± 2.49
3	17.77 ± 1.09	30.65 ± 1.33
T	NR	NR
2	16.06 ± 1.15	27.51 ± 1.24
4	NR	NR

^1^ D: diclofenac; HT: hydroxytyrosol; T: tyrosol; NR: not reached.

**Table 2:** Properties of nanomicelles in terms of size and drug content.

Formulation Type	Size (nm)	P.I.	Drug Content (µmol/mL)	Entrapment (%)	Loading (%)
Nano3Hybrid10	14.28 ± 0.24	0.2007	8.24 ± 0.29	80.32 ± 2.89	10.32 ± 0.37
Nano3Hybrid15	24.98 ± 0.46	0.3038	11.44 ± 0.27	79.41 ± 1.97	13.48 ± 0.33
Nano3Hybrid20	68.55 ± 5.09	0.3562	19.3 ± 0.54	96.48 ± 1.08	22.13 ± 0.34
Nano5Hybrid30	50.67 ± 5.2	0.3660	27.01 ± 0.58	93.51 ± 1.56	18.77 ± 0.31

**Table 3:** Permeation parameters: flux (J), lag time and amount of drug permeated through the skin (Qpermeated24h) at end of the experiment (24 h) from the formulations under study (mean ± SEM, *n* = 6).

Formulation	Drug	Flux, nmol/cm^2^·h	Lag time, h	Qpermeated 24 h, nmol
Solaraze	Diclofenac	2.01 ± 0.37	7.90 ± 0.50	32.53 ± 6.36
Nano3Hybrid10	Hybrid 1	No permeation		
	Diclofenac	0.12 ± 0.02	1.42 ± 0.80	3.49 ± 0.62
Nano3Hybrid15	Hybrid 1	No permeation		
	Diclofenac	0.04 ± 0.001	2.86 ± 1.57	1.18 ± 0.11
Nano3Hybrid20	Hybrid 1	No permeation		
	Diclofenac	0.58 ± 0.004	10.8 ± 0.03	9.42 ± 0.08
Nano5Hybrid30	Hybrid 1	0.16 ± 0.08	9.10 ± 3.76	3.27 ± 2.18
	Diclofenac	0.24 ± 0.06	7.09 ± 3.55	7.27 ± 4.32

**Table 4:** Amount of drugs permeated through and recovered into pig ear skin after application of formulations under study (nanomols, mean ± SEM, *n* = 6).

Formulations	Diclofenac Permeated	Diclofenac Recovered	Hybrid 1 Permeated	Hybrid 1 Recovered
Solaraze	32.53 ± 6.36	158 ± 20.5	n.a.	n.a.
Nano3Hybrid10	3.49 ± 0.62	1.47 ± 1.17	-	3.23 ± 0.33
Nano3Hybrid15	1.18 ± 0.11	1.68 ± 0.50	-	6.10 ± 0.97
Nano3Hybrid20	9.42 ± 0.08	28.21 ± 6.94	-	10.42 ± 0.92
Nano5Hybrid30	7.28 ± 4.32	18.96 ± 3.45	3.27 ± 2.19	14.1 ± 5.52

**Table 5:** Composition of the mixtures used for the preparation of hybrid-loaded nanomicelles.

Formulation Type	Total Surfactant (% *w*/*w*) *	Hybrid-1 (mM)
Nano3Hybrid10	3.0	10
Nano3Hybrid15	3.0	15
Nano3Hybrid20	3.0	20
Nano5Hybrid30	5.0	30

* Surfactant: mixture of Vitamin E-TPGS and OPEE (molar ratio 1:1).

## Supplementary Materials

The following are available online: the ^1^H-NMR and ^13^C-NMR spectra of the compounds **1**–**4**.
