# Supplementary material for: Diclofenac-Derived Hybrids for Treatment of Actinic Keratosis and Squamous Cell Carcinoma
Source: Molecules. 2019 May 9;24(9):1793. doi: 10.3390/molecules24091793 (PMC6539072; doi:10.3390/molecules24091793)
Supplement: Supplementary file 1 [file molecules-24-01793-s001.pdf]

Supporting information

1. NMR spectra

^{1}H NMR of **1**

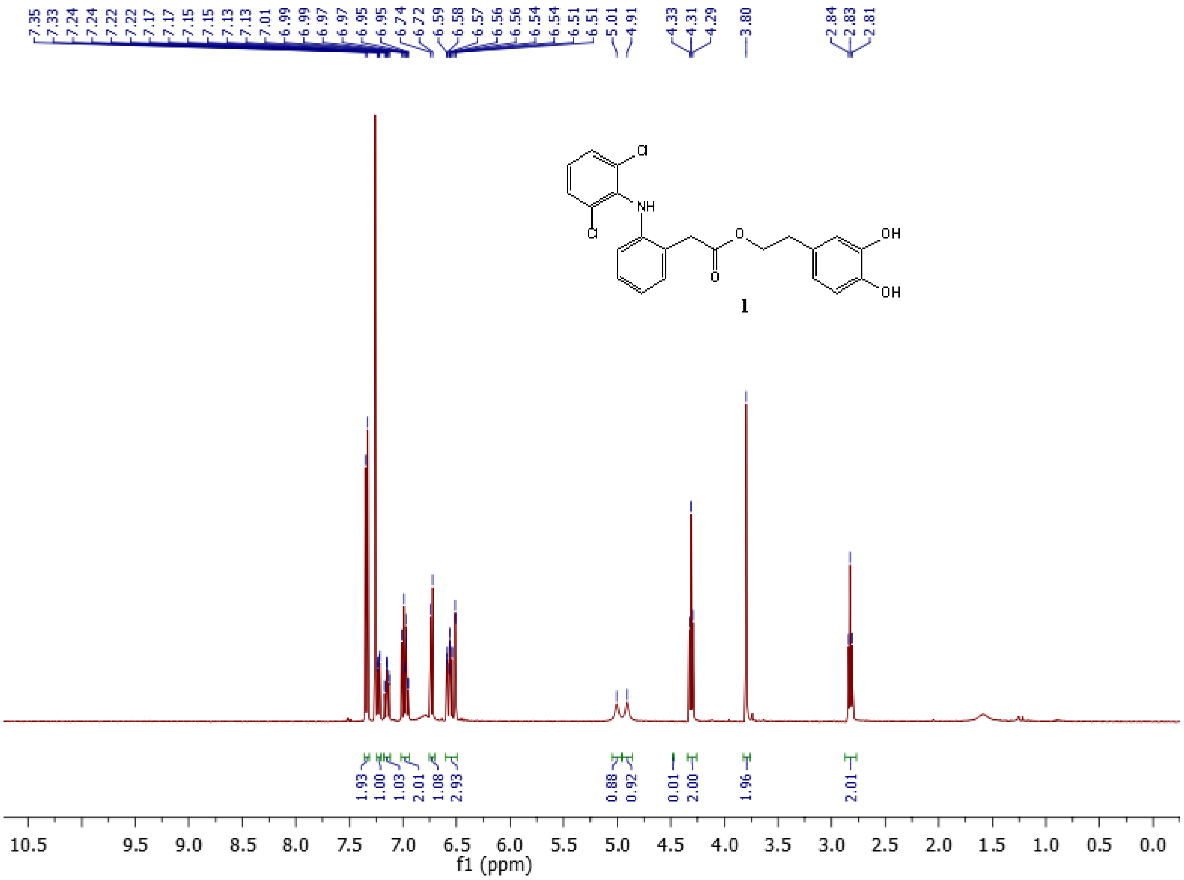

^{13}C NMR of **1**

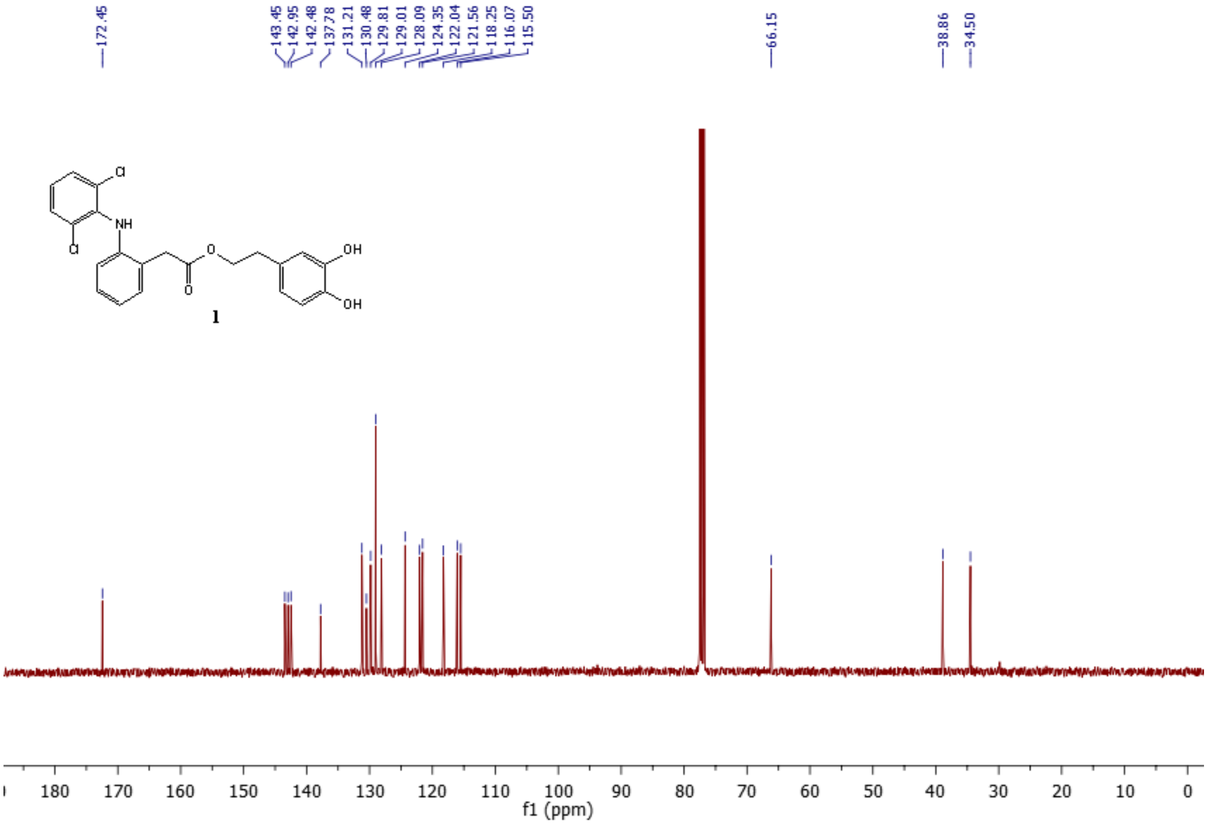
172.45
143.45
142.95
142.48
137.78
131.21
130.48
129.81
129.01
128.09
124.35
122.04
121.56
118.25
116.07
115.50
66.15
38.86
34.50
Cl
NH
Cl
O
OH
O
OH
1
180 170 160 150 140 130 120 110 100 90 80 70 60 50 40 30 20 10 0
f1 (ppm)

^{1}H NMR of **2**

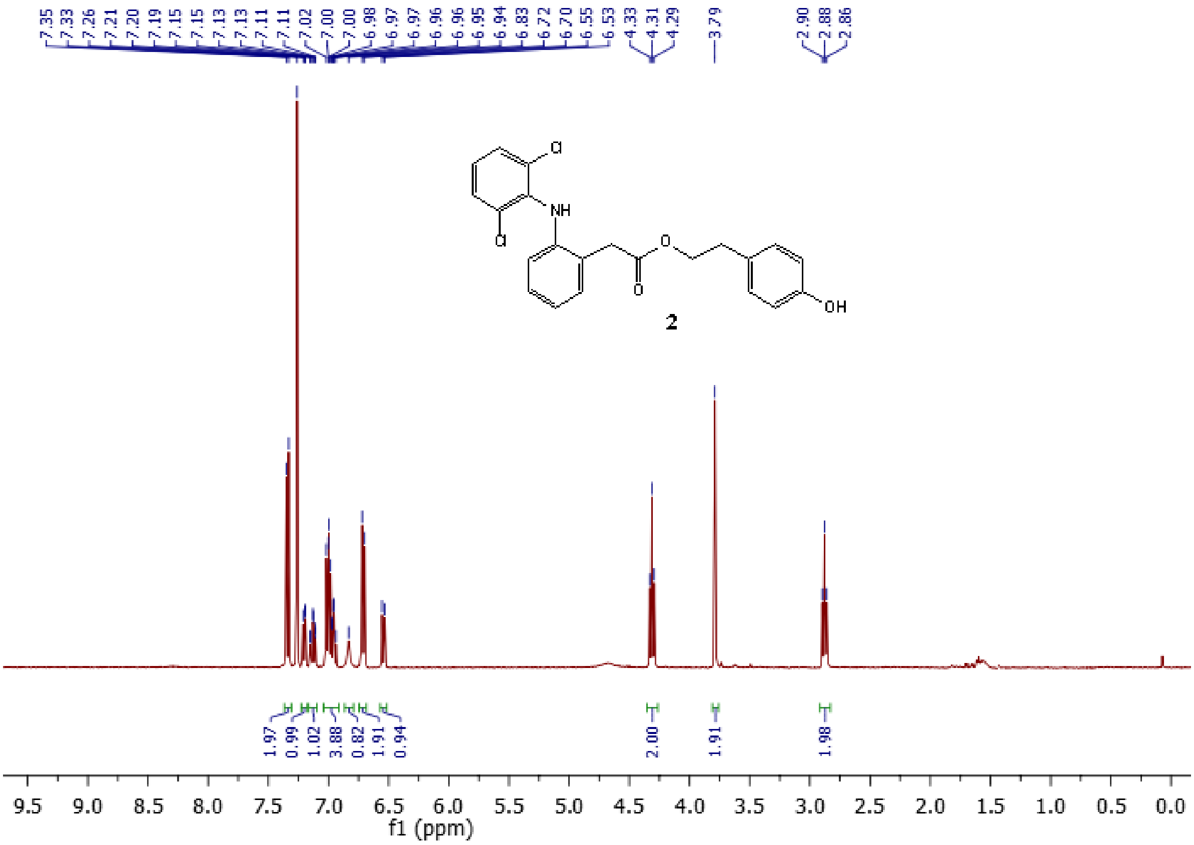
7.35
7.33
7.26
7.21
7.20
7.19
7.15
7.15
7.13
7.13
7.11
7.11
7.02
7.00
7.00
6.98
6.97
6.97
6.96
6.96
6.95
6.94
6.83
6.72
6.70
6.55
6.53
4.33
4.31
4.29
3.79
2.90
2.88
2.86
Cl
NH
Cl
O
O
OH
2
1.97
0.99
1.02
3.88
0.82
1.91
0.94
2.00
1.91
1.98
9.5
9.0
8.5
8.0
7.5
7.0
6.5
6.0
5.5
5.0
4.5
4.0
3.5
3.0
2.5
2.0
1.5
1.0
0.5
0.0
f1 (ppm)

^{13}C NMR of **2**

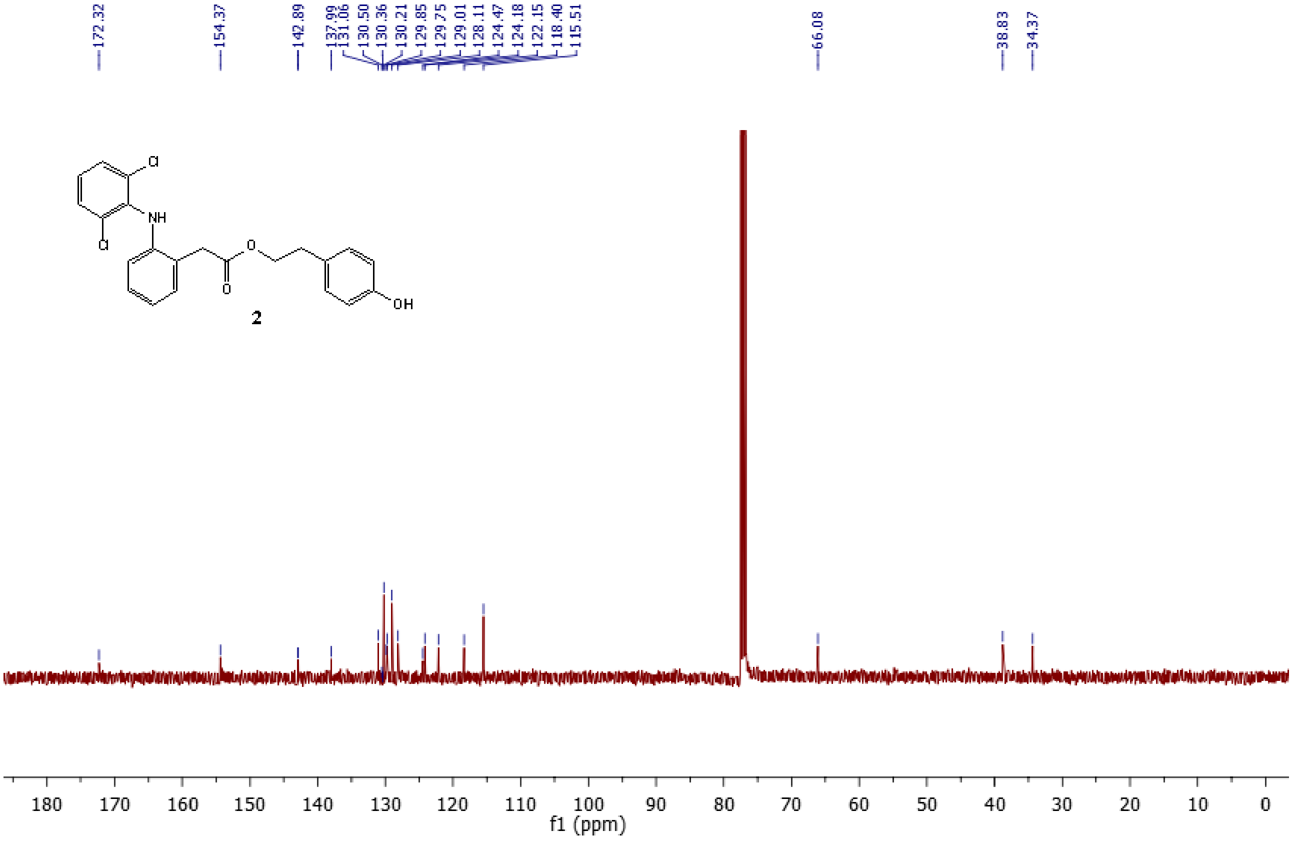
172.32
154.37
142.89
137.99
131.06
130.50
130.36
130.21
129.85
129.75
129.01
128.11
124.47
124.18
122.15
118.40
115.51
66.08
38.83
34.37
Cl
NH
Cl
O
O
OH
2
180 170 160 150 140 130 120 110 100 90 80 70 60 50 40 30 20 10 0
f1 (ppm)

^{1}H NMR of **3**

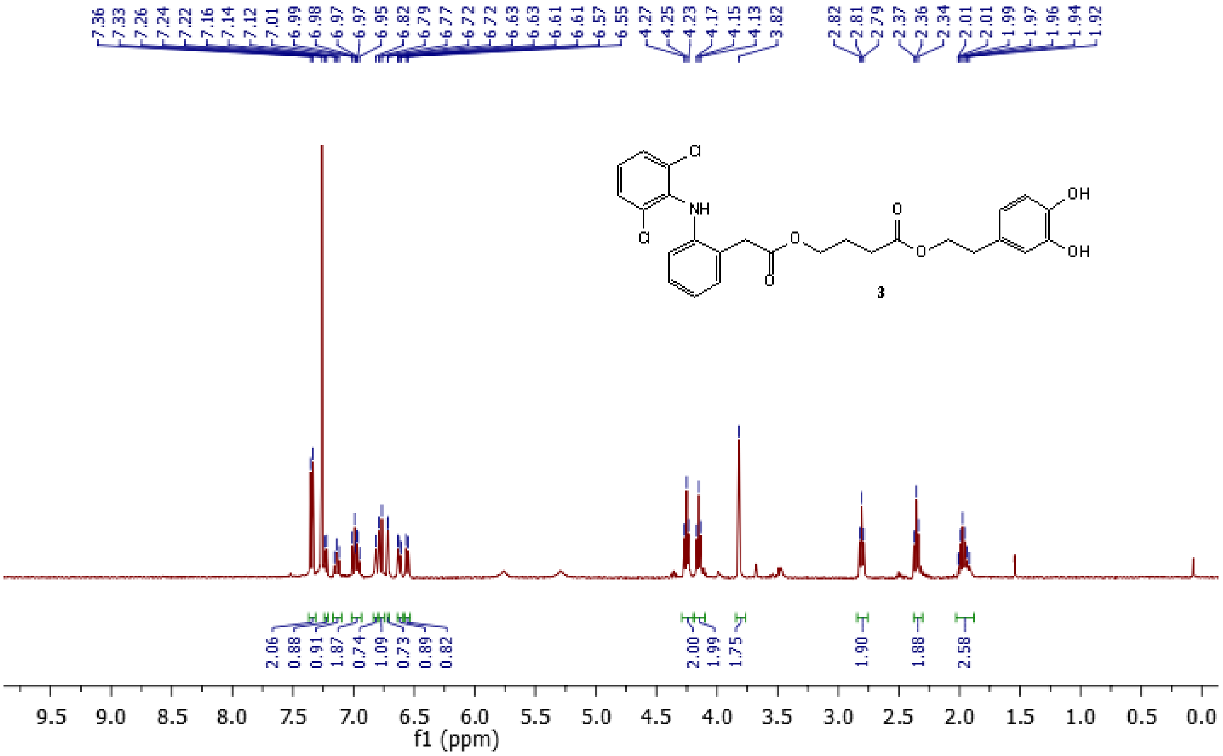

7.36 7.33 7.26 7.24 7.22 7.16 7.14 7.12 7.01 6.99 6.98 6.97 6.97 6.95 6.82 6.79 6.77 6.72 6.72 6.63 6.63 6.61 6.61 6.57 6.55 4.27 4.25 4.23 4.17 4.15 4.13 3.82
2.82 2.81 2.79 2.37 2.36 2.34 2.01 2.01 1.99 1.97 1.96 1.94 1.92
Cl
NH
Cl
O
O
O
O
OH
OH
3
2.06 0.88 0.91 1.87 0.74 1.09 0.73 0.89 0.82
2.00 1.99 1.75
1.90
1.88
2.58
9.5 9.0 8.5 8.0 7.5 7.0 6.5 6.0 5.5 5.0 4.5 4.0 3.5 3.0 2.5 2.0 1.5 1.0 0.5 0.0
f1 (ppm)

^{13}C NMR of **3**

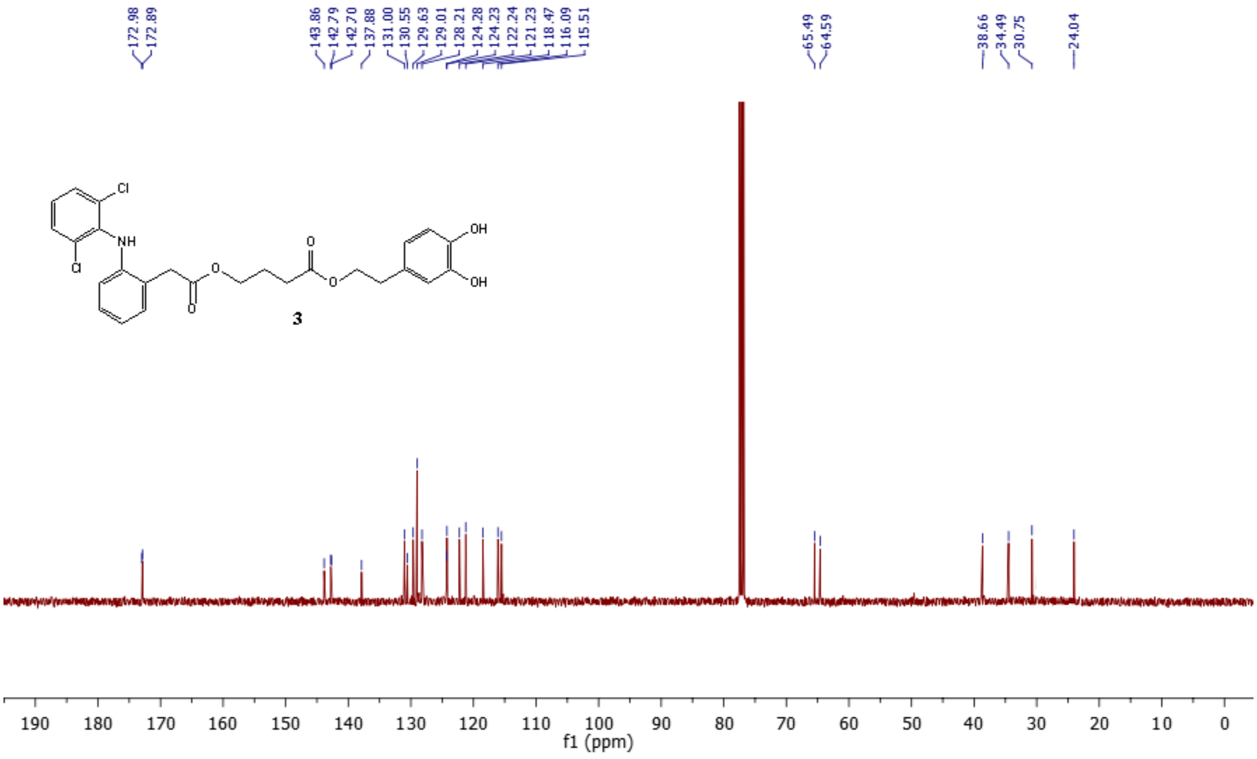

^{1}H NMR of **4**

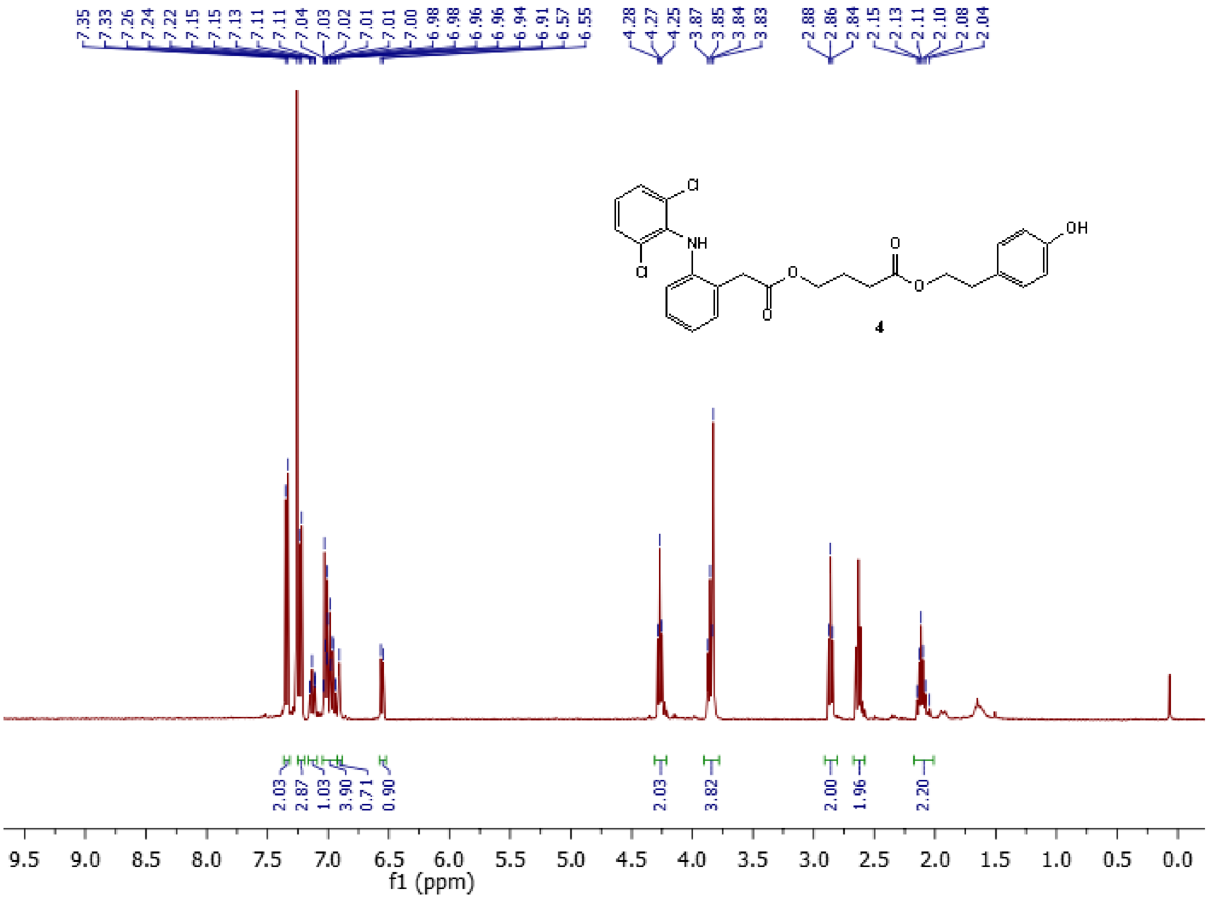
7.35
7.33
7.26
7.24
7.22
7.15
7.15
7.13
7.11
7.11
7.04
7.03
7.02
7.01
7.01
7.00
6.98
6.98
6.96
6.96
6.94
6.91
6.57
6.55
4.28
4.27
4.25
3.87
3.85
3.84
3.83
2.88
2.86
2.84
2.15
2.13
2.11
2.10
2.08
2.04
Cl
NH
Cl
O
O
O
O
O
OH
4
2.03
2.87
1.03
3.90
0.71
0.90
2.03
3.82
2.00
1.96
2.20
9.5
9.0
8.5
8.0
7.5
7.0
6.5
6.0
5.5
5.0
4.5
4.0
3.5
3.0
2.5
2.0
1.5
1.0
0.5
0.0
f1 (ppm)

^{13}C NMR of **4**

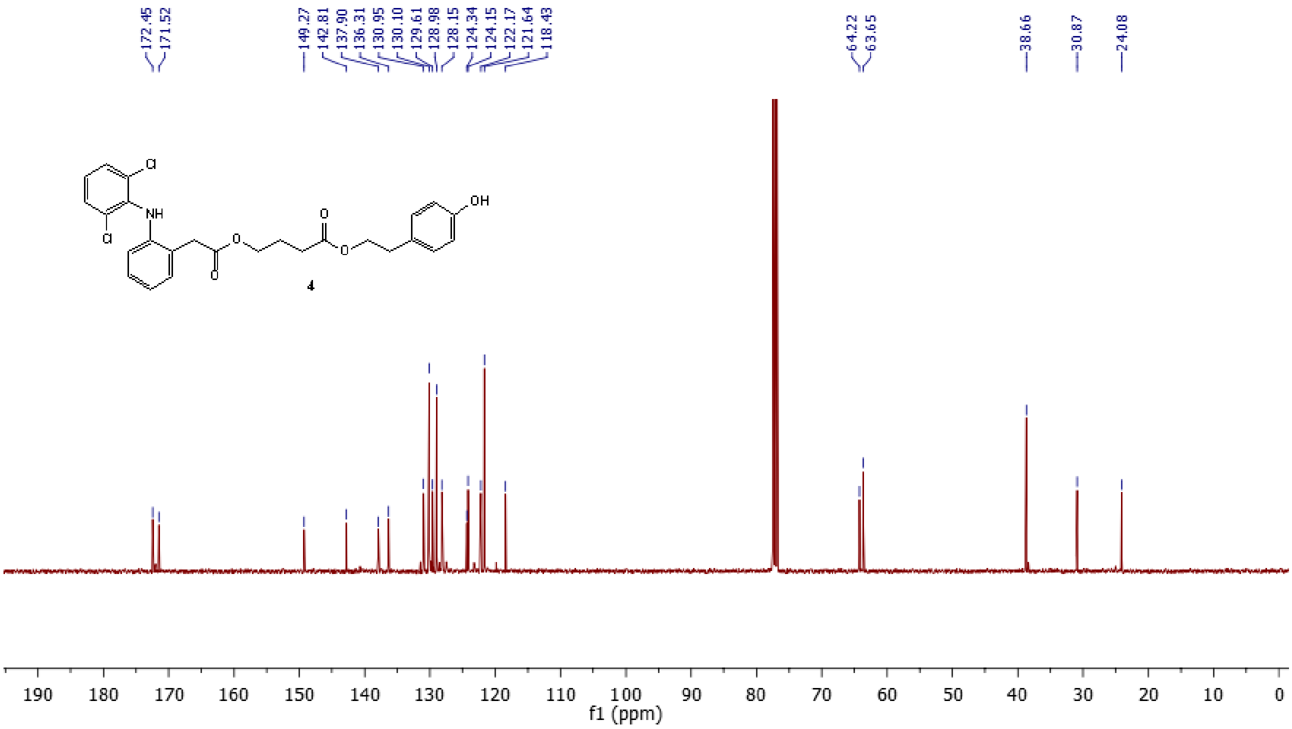

172.45
171.52
149.27
142.81
137.90
136.31
130.95
130.10
129.61
128.98
128.15
124.34
124.15
122.17
121.64
118.43
64.22
63.65
38.66
30.87
24.08
Cl
NH
Cl
O
O
O
O
OH
4
190
180
170
160
150
140
130
120
110
100
90
80
70
60
50
40
30
20
10
0
f1 (ppm)
